# Artificial Intelligence for Risk Prediction of Rehospitalization with Acute Kidney Injury in Sepsis Survivors

**DOI:** 10.3390/jpm12010043

**Published:** 2022-01-04

**Authors:** Shuo-Ming Ou, Kuo-Hua Lee, Ming-Tsun Tsai, Wei-Cheng Tseng, Yuan-Chia Chu, Der-Cherng Tarng

**Affiliations:** 1 Department of Medicine, Division of Nephrology, Taipei Veterans General Hospital, Taipei 11217, Taiwan; okokyytt@gmail.com (S.-M.O.); dadabim3520@gmail.com (K.-H.L.); mingtsun74@gmail.com (M.-T.T.); wctseng@gmail.com (W.-C.T.); 2 School of Medicine, National Yang Ming Chiao Tung University, Taipei 11221, Taiwan; 3 Institute of Clinical Medicine, National Yang Ming Chiao Tung University, Taipei 11221, Taiwan; 4 Center for Intelligent Drug Systems and Smart Bio-Devices (IDS2B), National Yang Ming Chiao Tung University, Hsinchu 30010, Taiwan; 5 Information Management Office, Taipei Veterans General Hospital, Taipei 11217, Taiwan; 6 Big Data Center, Taipei Veterans General Hospital, Taipei 11217, Taiwan; 7 Department of Information Management, National Taipei University of Nursing and Health Sciences, Taipei 11219, Taiwan; 8 Department and Institute of Physiology, National Yang Ming Chiao Tung University, Taipei 11221, Taiwan

**Keywords:** acute kidney injury, artificial intelligence, machine learning, rehospitalization, sepsis, sepsis survivors

## Abstract

Sepsis survivors have a higher risk of long-term complications. Acute kidney injury (AKI) may still be common among sepsis survivors after discharge from sepsis. Therefore, our study utilized an artificial-intelligence-based machine learning approach to predict future risks of rehospitalization with AKI between 1 January 2008 and 31 December 2018. We included a total of 23,761 patients aged ≥ 20 years who were admitted due to sepsis and survived to discharge. We adopted a machine learning method by using models based on logistic regression, random forest, extra tree classifier, gradient boosting decision tree (GBDT), extreme gradient boosting, and light gradient boosting machine (LGBM). The LGBM model exhibited the highest area under the receiver operating characteristic curves (AUCs) of 0.816 to predict rehospitalization with AKI in sepsis survivors and followed by the GBDT model with AUCs of 0.813. The top five most important features in the LGBM model were C-reactive protein, white blood cell counts, use of inotropes, blood urea nitrogen and use of diuretics. We established machine learning models for the prediction of the risk of rehospitalization with AKI in sepsis survivors, and the machine learning model may set the stage for the broader use of clinical features in healthcare.

## 1. Introduction

Sepsis is estimated to affect 19.4 million patients, with an annual sepsis-related mortality of approximately 5.3 million cases [1]. Therefore, sepsis is a major public health concern due to the life-threatening organ dysfunction and the dysregulated host response to infection, and sepsis is a common cause of death in hospitalized patients [2,3]. As there has been significant medical progress in decreasing mortality and morbidity after sepsis, attention to the complications after discharge in sepsis survivors has become more important [4,5,6,7].

Acute kidney injury (AKI) frequently occurs with sepsis due to pathologic interactions of multiple organ dysfunction, systemic hypotension, inflammatory cytokine storms and nephrotoxic drugs, which all indirectly and directly contributing to renal injury [8,9,10]. Previous studies have found that 40% to 50% of patients with AKI had sepsis [8,11], and approximately 11% to 42% of patients with sepsis developed AKI [12,13,14]. Unplanned rehospitalization is associated with worsening patient outcomes and increased treatment costs [15,16]. Although AKI is a common complication in sepsis, the risks of rehospitalization with AKI in sepsis survivors remains unknown. Therefore, the development of a prediction model for rehospitalization with AKI has become an important therapeutic goal in the management of sepsis survivors.

To appropriately manage rehospitalization with AKI in sepsis survivors, a precise prediction model for identifying high-risk patients is required to optimize the treatment strategy. This predictive model is important not only to allow a more comprehensive prognostication of patients’ well-being but also to reduce the healthcare financial burdens. Machine learning models have already been applied in many fields, such as outcome prediction [17,18,19], and these models may potentially be used to identify high-risk patients. Machine learning models have been mostly described to predict episodes of the occurrence of AKI during sepsis [20,21,22]. However, there is no study to evaluate their effects on rehospitalization with AKI after patients who survived to discharge from sepsis. To resolve this important issue, we conducted a large-scale cohort study of sepsis survivors, and the predictive ability of the machine learning model was compared to select the optimal machine learning model.

## 2. Materials and Methods

### 2.1. Study Design and Data Source

We established a database including the detailed information of sepsis survivors extracted from the Big Data Center of Taipei Veterans General Hospitals between 1 January 2008 and 31 December 2018, which included the comprehensive medical records from the inpatient, outpatient, and emergent departmental records [23]. The detailed patient demographic, clinical, diagnostic/procedural information, drug prescriptions, procedural codes, and laboratory data were included in our analysis. To identify the sepsis survivors, we included all patients with discharge codes based on the International Classification of Diseases, Ninth and Tenth Edition, Clinical Modification (ICD-9-CM and ICD-10-CM) codes for sepsis (ICD code 038, 995.91, A40 and A41), severe sepsis (ICD code 995.92 and R65.20) or septic shock (ICD code 785.52 and R65.21) during the study period who were discharged alive [24]. We excluded patients who had pre-existing end-stage kidney disease maintained with dialysis or kidney transplant before discharge, were younger than 20 years old, or who died during admission.

### 2.2. Class Definition

The class was labeled as 1 if there was rehospitalization with AKI during the follow-up periods; otherwise, the class was labeled as 0 if there was no rehospitalization with AKI. The diagnosis of AKI was defined as a 0.3 mg/dL within 48 h or 50% increase within 7 days from the baseline creatinine based on the Kidney Disease Improving Global Outcomes classification (KDIGO) definition [25]. We included the first-time rehospitalization with AKI because multiple admissions may introduce a bias favoring survivors.

### 2.3. Machine Learning Algorithm and Statistical Analysis

Continuous data are presented as the median (interquartile ranges (IQRs)) and categorical data are presented as numbers (proportions). Before the machine learning processes, the missing values of the clinical variables were imputed using the k-nearest neighbors (KNN) algorithm [26,27]. The whole dataset was then randomly split into a training dataset and a validation dataset at a ratio of 70:30%, respectively. In our study, we used several machine learning methods, including logistic regression, a random forest [28], an extra tree classifier [29], an extreme gradient boosting (XGBoost) [30], a light gradient boosting machine (LGBM) [31], and a gradient boosting decision tree (GBDT) [32], to predict risks of rehospitalization with AKI. The prediction abilities of various machine learning models were examined based on the area under the curve of receiver operating characteristics (AUCs) and precision-recall curves of each model. As the methods of prediction in machine learning models are often unclear, we used SHapley Additive exPlanation (SHAP) values to provide accurate attribution values for each clinical feature in our prediction model [33,34,35]. The data were analyzed by using Python (Python Software Foundation version 3.7.6, available at http://www.python.org, accessed on 1 November 2021). All tests were two-tailed, and a *p* value < 0.05 was statistically significant.

## 3. Results

### 3.1. Study Population

In the 10-year study period, 23,761 sepsis survivors were included in our final cohort, and the detailed patient demographic data are presented in Table 1. Sepsis survivors were predominantly female, and 55.7% of the patients had hypertension, 32.8% had diabetes mellites, and 44.3% used CCBs. The patients had a baseline creatinine level of 1.1 mg/dL. We further divided the sepsis survivors randomly into the two groups and allocated 70% of them to the training set and the remaining 30% to the test set. Among these patients, 8756 (36.9%) sepsis survivors had episodes of rehospitalization with AKI in sepsis survivors with the median intervals from discharge to rehospitalization of 8.6 months. In addition, there were 6076 (36.5%) and 2680 (37.6%) episodes of rehospitalization with AKI in the training and testing datasets, respectively.

### 3.2. Model Prediction Ability

**Figure 1:**
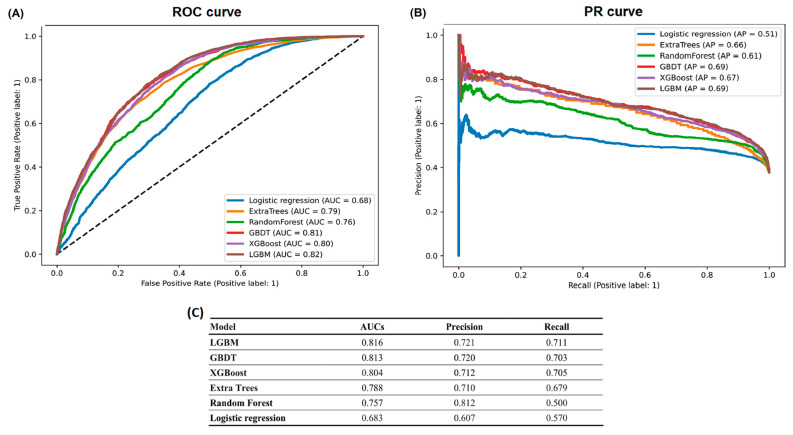
Comparison of (**A**) ROC curve, (**B**) PR curve and (**C**) predictive ability among different machine learning models. Abbreviations: ROC, receiver operating characteristic curve; PR, precision-recall; GBDT, gradient boosting decision tree; XGBoost, extreme gradient boosting; LGBM, light gradient boosting machine LGBM. Abbreviations: ROC, receiver operating characteristic curve; PR, precision-recall; AUC, area under the curve of receiver operating characteristic curve; LGBM, light gradient boosting machine; GBDT, Gradient Boosting Decision Tree; XGBoost, extreme gradient boosting.

The 84 features, including the demographic characteristics, underlying comorbidities, laboratory data and concomitant medications, that were used in our machine learning models are listed in Table 2. We utilized the following machine learning methods with all the clinical features as input variables: logistic regression, random forest, extra tree classifier, XGBoost, GBDT, and LGBM (Figure 1). Regarding the predictive ability of the models for outcome prediction, the LGBM exhibited the largest AUC of 0.816, and the GBDT model had the second highest AUC of 0.813. The logistic regression exhibited the smallest AUC of 0.683.

### 3.3. Ranks of Feature Importance and SHAP Value in the Machine Learning Models

**Figure 2:**
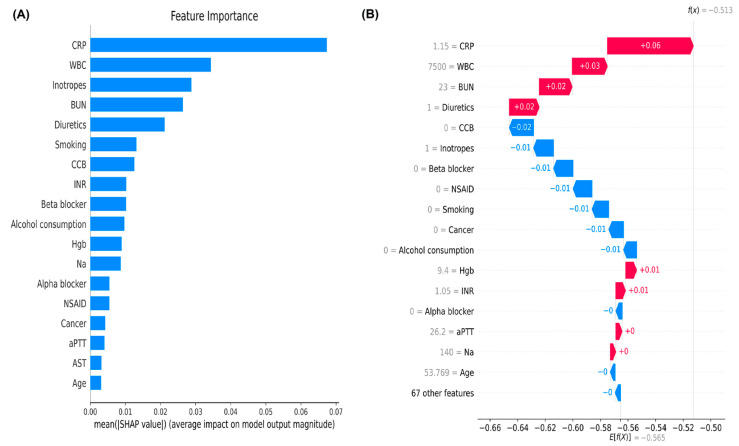
(**A**) The feature importance plot using SHAP value reveals the important features in the final LGBM model (**B**)The local bar plots of feature importance reveal the SHAP values for features in the model. The feature values are show in gray to the left of the feature names. Abbreviations: SHAP, SHapley Additive exPlanation; LGBM, light gradient boosting machine; CRP, C-reactive protein; WBC, white blood cell counts; BUN, blood urea nitrogen; CCB, calcium channel blocker; INR, international normalized ratio; Hgb, hemoglobin; Na, sodium; NSAID, non-steroidal anti-inflammatory drug; aPTT, activated partial thromboplastin time; AST, aspartate aminotransferase; INR, international normalized ratio.

**Figure 3:**
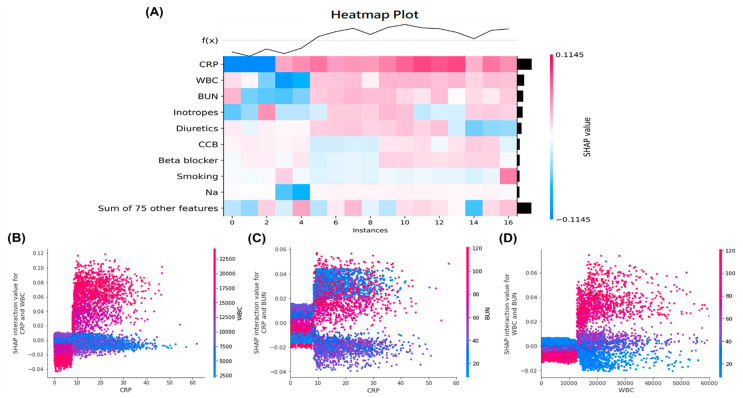
(**A**) The SHAP heatmap plot with the model’s predictions for important features. The heatmap plot function creates a plot with the features on the y-axis, the model inputs on the x-axis, and the SHAP values encoded on a color scale with a higher value being redder, and a lower value being bluer. The SHAP dependence plots showed the interaction effects between (**B**) CRP and WBC, (**C**) CRP and BUN and (**D**) WBC and BUN in the LGBM model. Abbreviations: SHAP, SHapley Additive exPlanation; CRP, C-reactive protein; WBC, white blood cell counts; BUN, blood urea nitrogen; LGBM, light gradient boosting machine.

To identify important features in the LGBM model, we performed a feature importance plot by using SHAP values and listed the features in descending order. The top five important features were C-reactive protein, white blood cell counts, use of inotropes, blood urea nitrogen, and use of diuretics, which contribute to higher predictive powers than the bottom features (Figure 2A). The local bar plot of a sepsis survivor showed how the SHAP values of features affected the model prediction (Figure 2B). Red SHAP values increased the prediction, and blue values decreased it. The SHAP heatmap plot were shown in Figure 3A, and features with higher SHAP values were highlighted in redder boxes. The dependent plots revealed the interaction effects between C-reactive protein (which is the top-most important feature), white blood cell counts, and blood urea nitrogen in our LGBM model (Figure 3B–D).

## 4. Discussion

In our cohort study, 23,761 sepsis survivors suffered from rehospitalization with AKI after discharge. We developed machine learning algorithms using 84 clinical features to predict rehospitalization with AKI and compared the AUCs of the different machine learning models. We found that the LGBM model had the highest AUC of 0.816 compared to the other machine learning models. Our study suggests that AKI might still be an unrecognized outcome after discharge from sepsis, and the use of machine learning models may help to predict rehospitalization with AKI.

AKI is frequently observed in patients with sepsis, and a study including 2871 patients from the critical care database developed risk-prediction nomogram for AKI with C-index of 0.75 [20]. Another study including 15,726 patients with sepsis from the same critical care database established a prediction model by using logistic regression with a C-index of 0.71 [21]. The prediction models established by these studies were limited by only using the logistic regression method rather than other machine learning models to improve the predictive ability. Moreover, a study including 5984 septic patients with AKI established five prediction models, including logistic regression, random forest, support vector machine, artificial neural network, and extreme gradient boosting to predict persistent AKI [22]. The artificial neural network and logistic regression models achieved the highest AUC of 0.76. However, none of the studies carried out so far have considered whether sepsis survivors are still at greater risks of AKI after discharge.

Sepsis survivors were found to be the highest risks for short- and long-term outcomes after discharge from sepsis [7,36,37,38]. However, the rates of rehospitalization with AKI have never been explored, and an understanding of such complication is required for physicians to initiate early treatment and follow-up strategies. In our study, the incidence of rehospitalization with AKI is still high, with approximately 36.9% of sepsis survivors after discharge. Our study is the first in the literature to use a machine learning approach to predict risks of rehospitalization with AKI, and the optimal AUC was achieved to 0.816 in the LGBM model. The performance of LGBM was higher than that of traditional logistic regression model (AUC: 0.683) for the prediction of rehospitalization with AKI.

In addition, the feature importance plot using SHAP value in our LGBM found some important predictors for risks of rehospitalization with AKI, some of which were consistent with the traditional factors. The important predictors for AKI, such as C-reactive protein, white blood cell counts, and the use of inotropes may be associated with the infectious status before discharge. In addition, blood urea nitrogen levels and the use of diuretics may reflex the fluid status and were traditionally associated with future risks of rehospitalization with AKI. Therefore, our machine learning model may help identify high-risk sepsis survivors who are prone to rehospitalization with AKI after considering clinical features related to their infection conditions or fluid status.

Our study has several strengths. First, compared to previous studies, our study is the first to predict the risks of rehospitalization with AKI after discharge from sepsis for a large number of sepsis survivors. Second, our study evaluated the laboratory data, and we included sepsis survivors who had more than two serum creatinine measurements. Therefore, we had the ability to discriminate the sepsis survivors’ outcomes, including the rehospitalization with AKI, which may reduce the possible underreporting or misclassifications of AKI by using the International Statistical Classification of Diseases and Related Health Problems (ICD) coding, compared to other studies that extracted data from administrative datasets. Finally, we established predictive models of machine learning algorithms that might be important to apply in clinical practice.

Our study may have several limitations that should be noted. First, because of the nature of observational studies, the causal inference of rehospitalization with AKI might be confounded by unmeasured factors. Second, as our analysis was based on a single tertiary medical center’s data, some age and disease group particularities regarding old age (median, 76.4 years) and higher cancer incidence (48.8%), which are factors that may induce some bias to the renal function or rehospitalization with AKI in the analyzed subjects. Third, the machine learning algorithm learned from the input clinical features, and some hidden relationships may be unknown if the features were not included by the physicians.

## 5. Conclusions

Our study established a machine learning algorithm for the detection and prediction of rehospitalization with AKI. Therefore, our findings support the implementation of a useful machine learning algorithm for risks of rehospitalization with AKI. Due to the age distribution, disease particularities, and single-center-based character of our study, external validation is required to evaluate the generalizability.

## Figures and Tables

**Table 1:** Clinical features and classes in sepsis survivors divided into training and testing dataset.

	All Patients	Training Set	Testing Set
	(*n* = 23,761)	(*n* = 16,632)	(*n* = 7129)
Demographic and clinical characteristics			
Age, years	76.4 (61.4, 85.2)	76.4 (61.2, 85.2)	76.4 (61.9, 85.2)
Male sex, *n* (%)	8557 (36.0)	5995 (36.0)	2562 (35.9)
Smoking, *n* (%)	5373 (22.6)	3744 (22.5)	1629 (22.9)
Alcohol consumption, *n* (%)	3945 (16.6)	2739 (16.5)	1206 (16.9)
Underlying Comorbidities			
Hypertension, *n* (%)	13,238 (55.7)	9271 (55.7)	3967 (55.6)
Transient ischemic attack, *n* (%)	579 (2.4)	399 (2.4)	180 (2.5)
Ischemic stroke, *n* (%)	3343 (14.1)	2358 (14.2)	985 (13.8)
Hemorrhagic stroke, *n* (%)	1084 (4.6)	774 (4.7)	310 (4.3)
Dementia, *n* (%)	3190 (13.4)	2218 (13.3)	972 (13.6)
Diabetes mellitus, *n* (%)	7803 (32.8)	5432 (32.7)	2371 (33.3)
Gout, *n* (%)	2443 (10.3)	1737 (10.4)	706 (9.9)
Myocardial infarction, *n* (%)	1852 (7.8)	1272 (7.6)	580 (8.1)
Coronary artery disease, *n* (%)	6260 (26.3)	4308 (25.9)	1952 (27.4)
CHF, *n* (%)	4759 (20.0)	3282 (19.7)	1477 (20.7)
Atrial fibrillation, *n* (%)	2394 (10.1)	1667 (10.0)	727 (10.2)
Chronic liver disease, *n* (%)	3875 (16.3)	2729 (16.4)	1146 (16.1)
Cirrhosis, *n* (%)	1395 (5.9)	996 (6.0)	399 (5.6)
Peptic ulcer disease, *n* (%)	5632 (23.7)	3957 (23.8)	1675 (23.5)
COPD, *n* (%)	4469 (18.8)	3110 (18.7)	1359 (19.1)
Asthma, *n* (%)	1192 (5.0)	835 (5.0)	357 (5.0)
PAOD, *n* (%)	192 (0.8)	130 (0.8)	62 (0.9)
Autoimmune disease, *n* (%)	821 (3.5)	591 (3.6)	230 (3.2)
Cancer, *n* (%)	11,592 (48.8)	8145 (49.0)	3447 (48.4)
Valvular heart disease, *n* (%)	1303 (5.5)	908 (5.5)	395 (5.5)
Critical conditions			
ICU admission, *n* (%)	12,962 (54.6)	9041 (54.4)	3921 (55.0)
Use of mechanical ventilators, *n* (%)	8740 (36.8)	6083 (36.6)	2657 (37.3)
Use of inotropes, *n* (%)	11,343 (47.7)	7933 (47.7)	3410 (47.8)
Laboratory data			
Blood urea nitrogen, mg/dL	24.0 (14.0, 51.0)	24.0 (14.0, 51.0)	24.0 (14.0, 50.0)
Creatinine, mg/dL	1.1 (0.7, 2.1)	1.1 (0.7, 2.2)	1.1 (0.7, 2.1)
White blood cells, /mm^3^	8100 (5700, 11,900)	8100 (5700, 11,900)	8100 (5700, 12,000)
Hemoglobin, g/dL	10.1 (8.9, 11.5)	10.1 (8.9, 11.5)	10.1 (9.0, 11.6)
Sodium, mmol/L	139.0 (135.0, 142.0)	139.0 (135.0, 142.0)	139.0 (135.0, 142.0)
Potassium, mmol/L	4.1 (3.6, 4.6)	4.1 (3.6, 4.6)	4.1 (3.6, 4.6)
Chloride, mmol/L	103.0 (98.0, 106.0)	103.0 (98.0, 106.0)	103.0 (98.0, 106.0)
Calcium, mg/dL	8.5 (8.0, 9.0)	8.5 (8.0, 9.0)	8.5 (8.0, 9.0)
Phosphate, mg/dL	3.3 (2.6, 4.0)	3.3 (2.6, 4.0)	3.3 (2.7, 4.1)
HCO_3_, mmol/L	23.7 (19.3, 28.0)	23.7 (19.3, 28.0)	23.8 (19.4, 28.0)
C-reactive protein, mg/dL	3.4 (1.2, 9.0)	3.4 (1.2, 9.1)	3.3 (1.1, 8.7)
Albumin, mg/dL	3.0 (2.6, 3.4)	3.0 (2.6, 3.4)	3.0 (2.6, 3.4)
Alanine transaminase, U/L	25.0 (15.0, 44.0)	25.0 (15.0, 45.0)	25.0 (15.0, 44.0)
Aspartate transaminase, U/L	29.0 (20.0, 51.0)	29.0 (20.0, 51.0)	29.0 (20.0, 50.0)
Alkaline phosphatase, U/L	95.0 (70.0, 147.0)	95.0 (69.0, 147.0)	94.0 (70.0, 147.0)
Gamma-glutamyl transferase, U/L	54.0 (25.0, 125.0)	53.0 (25.0, 125.0)	54.0 (24.0, 126.0)
Total bilirubin, mg/dL	0.6 (0.4, 1.1)	0.6 (0.4, 1.1)	0.6 (0.4, 1.1)
HbA_1c_, %	6.4 (5.8, 7.4)	6.4 (5.8, 7.4)	6.4 (5.8, 7.4)
Glucose, mg/dL	116.0 (95.0, 156.0)	116.0 (94.0, 155.0)	117.0 (95.0, 157.0)
Uric acid, mg/dL	5.5 (4.1, 7.1)	5.5 (4.1, 7.1)	5.6 (4.1, 7.1)
Cholesterol, mg/dL	151.0 (122.0, 182.0)	152.0 (122.0, 183.0)	150.0 (121.0, 181.0)
LDL-C, mg/dL	91.0 (70.0, 114.0)	91.0 (70.0, 115.0)	91.0 (69.0, 113.0)
HDL-C, mg/dL	41.0 (32.0, 51.0)	41.0 (32.0, 51.0)	41.0 (32.0, 51.0)
INR	1.1 (1.0, 1.2)	1.1 (1.0, 1.2)	1.1 (1.0, 1.2)
aPTT, seconds	29.9 (27.1, 34.0)	29.9 (27.2, 34.2)	29.9 (27.1, 33.8)
D-Dimer, ug/mL	3.6 (1.6, 8.1)	3.6 (1.5, 7.7)	3.9 (1.8, 9.3)
LDH, U/L	253.0 (196.0, 361.0)	252.0 (196.0, 361.0)	255.0 (197.0, 361.0)
NT-proBNP, pg/mL	3146.0 (836.5, 11,617.0)	3142.0 (823.8, 11,648.5)	3185.0 (856.8, 11,580.8)
Concomitant Medications			
ACEI, *n* (%)	2225 (9.4)	1537 (9.2)	688 (9.7)
ARB, *n* (%)	6972 (29.3)	4884 (29.4)	2088 (29.3)
Alpha, blocker, *n* (%)	6109 (25.7)	4228 (25.4)	1881 (26.4)
Beta blocker, *n* (%)	8521 (35.9)	5891 (35.4)	2630 (36.9)
CCB, *n* (%)	10,534 (44.3)	7362 (44.3)	3172 (44.5)
Warfarin, *n* (%)	1263 (5.3)	893 (5.4)	370 (5.2)
DOAC, *n* (%)	147 (0.6)	106 (0.6)	41 (0.6)
Aspirin, *n* (%)	5445 (22.9)	3743 (22.5)	1702 (23.9)
Plavix, *n* (%)	3267 (13.7)	2242 (13.5)	1025 (14.4)
Nitrate, *n* (%)	6521 (27.4)	4473 (26.9)	2048 (28.7)
Statin, *n* (%)	3446 (14.5)	2387 (14.4)	1059 (14.9)
Diuretic, *n* (%)	14,714 (61.9)	10,287 (61.9)	4427 (62.1)
Spironolactone, *n* (%)	4927 (20.7)	3427 (20.6)	1500 (21.0)
Metformin, *n* (%)	3459 (14.6)	2447 (14.7)	1012 (14.2)
Sulfonylurea, *n* (%)	2214 (9.3)	1533 (9.2)	681 (9.6)
Meglitinide, *n* (%)	2150 (9.0)	1495 (9.0)	655 (9.2)
SGLT2 inhibitor, *n* (%)	47 (0.2)	33 (0.2)	14 (0.2)
GLP1 receptor agonist, *n* (%)	3 (0.0)	3 (0.0)	0 (0.0)
Dipeptidyl peptidase-4 inhibitor, *n* (%)	2720 (11.4)	1883 (11.3)	837 (11.7)
Thiazolidinedione, *n* (%)	283 (1.2)	203 (1.2)	80 (1.1)
Alpha-glucosidase inhibitor, *n* (%)	1084 (4.6)	744 (4.5)	340 (4.8)
Insulin, *n* (%)	11,163 (47.0)	7810 (47.0)	3353 (47.0)
NSAID, *n* (%)	11,300 (47.6)	7917 (47.6)	3383 (47.5)
COX-2 inhibitor, *n* (%)	3316 (14.0)	2284 (13.7)	1032 (14.5)
Proton pump inhibitor, *n* (%)	13,642 (57.4)	9506 (57.2)	4136 (58.0)
Steroid, *n* (%)	8227 (34.6)	5781 (34.8)	2446 (34.3)
Allopurinol, *n* (%)	1583 (6.7)	1110 (6.7)	473 (6.6)
Febuxostat, *n* (%)	1446 (6.1)	1006 (6.0)	440 (6.2)
Benzbromarone, *n* (%)	1424 (6.0)	1007 (6.1)	417 (5.8)
Class/Outcome			
Rehospitalization with AKI ^†^	8756 (36.9)	6076 (36.5)	2680 (37.6)

Data are presented as *n* (%) or median and interquartile range. ^†^ AKI was defined based on the criteria from Kidney Disease: Improving Global Outcomes. Abbreviations: CHF, congestive heart failure; COPD, chronic obstructive pulmonary disease; PAOD, peripheral arterial occlusive disease; ICU, intensive care unit; HCO_3_, bicarbonate; HbA_1c_, hemoglobin A1c; LDL-C, low-density lipoprotein cholesterol; HDL-C, high-density lipoprotein-cholesterol; INR, international normalized ratio; aPTT, activated partial thromboplastin time; LDH, lactate dehydrogenase; NT-proBNP, N-terminal pro-brain natriuretic peptide; ACEI, angiotensin converting enzyme inhibitors; ARB, angiotensin receptor blocker; CCB, calcium channel blocker; DOAC, direct oral anticoagulant; SGLT2, sodium-glucose cotransporter 2 inhibitor; GLP1, glucagon-like peptide-1; NSAID, nonsteroidal anti-inflammatory drug; COX-2, cyclooxygenase-2; AKI, acute kidney injury.

**Table 2:** Clinical features selected in machine learning algorithm.

Demographics	Comorbidities	Laboratory Data	Containment Medications
Age	Hypertension	Blood urea nitrogen	ACEI
Gender	Transient ischemic attack	Creatinine	ARB
Smoking	Ischemic stroke	White blood cell counts	Alpha blocker
Alcohol consumption	Hemorrhagic stroke	Hemoglobin	Beta blocker
	Dementia	Sodium	CCB
	Diabetes mellitus	Potassium	Warfarin
	Gout	Chloride	DOAC
	Myocardial infarction	Calcium	Aspirin
	Coronary artery disease	Phosphate	Plavix
	CHF	HCO_3_	Nitrate
	Atrial fibrillation	C-reactive protein	Statin
	Chronic liver disease	Albumin	Diuretic
	Cirrhosis	Alanine transaminase	Spironolactone
	Peptic ulcer disease	Aspartate transaminase	Metformin
	COPD	Alkaline phosphatase	Sulfonylurea
	Asthma	Gamma-glutamyl transferase	Meglitinide
	PAOD	Total bilirubin	SGLT2 inhibitor
	Autoimmune disease	HbA_1c_	GLP1 receptor agonist
	Cancer	Glucose	DPP4 inhibitor
	Valvular heart disease	Uric acid	Thiazolidinedione
	ICU admission	Cholesterol	Alpha-glucosidase inhibitor
	Use of mechanical ventilators	LDL-C	Insulin
	Use of inotropes	HDL-C	NSAID
		INR	COX-2 inhibitor
		aPTT	Proton pump inhibitor
		D-dimer	Steroid
		LDH	Allopurinol
		NT-proBNP	Febuxostat
			Benzbromarone

Abbreviations: CHF, congestive heart failure; COPD, chronic obstructive pulmonary disease; PAOD, peripheral arterial occlusive disease; ICU, intensive care unit; HCO_3_, bicarbonate; HbA_1c_, hemoglobin A1c; LDL-C, low-density lipoprotein cholesterol; HDL-C, high-density lipoprotein-cholesterol; INR, international normalized ratio; aPTT, activated partial thromboplastin time; LDH, lactate dehydrogenase; NT-proBNP, N-terminal pro-brain natriuretic peptide; ACEI, angiotensin converting enzyme inhibitors; ARB, angiotensin receptor blocker; CCB, calcium channel blocker; DOAC, direct oral anticoagulant; SGLT2, sodium-glucose cotransporter 2 inhibitor; GLP1, glucagon-like peptide-1; DPP4, Dipeptidyl peptidase-4; NSAID, nonsteroidal anti-inflammatory drug; COX-2, cyclooxygenase-2.

## Data Availability

The data analyzed in this study are not publicly available because individual privacy may be compromised. Interested groups could contact Shuo-Ming Ou at okokyytt@gmail.com to request permission to access these datasets.
